# Highly Sensitive Determination of Hydrogen Peroxide and Glucose by Fluorescence Correlation Spectroscopy

**DOI:** 10.1371/journal.pone.0022955

**Published:** 2011-08-05

**Authors:** Satoshi Watabe, Yuki Sakamoto, Mika Morikawa, Ryuichi Okada, Toshiaki Miura, Etsuro Ito

**Affiliations:** 1 BL Co. Ltd., Numazu, Shizuoka, Japan; 2 Kagawa School of Pharmaceutical Sciences, Tokushima Bunri University, Sanuki, Kagawa, Japan; 3 Graduate School of Pharmaceutical Sciences, Hokkaido University, Kita-ku, Sapporo, Hokkaido, Japan; Stanford, United States of America

## Abstract

**Background:**

Because H_2_O_2_ is generated by various oxidase-catalyzed reactions, a highly sensitive determination method of H_2_O_2_ is applicable to measurements of low levels of various oxidases and their substrates such as glucose, lactate, glutamate, urate, xanthine, choline, cholesterol and NADPH. We propose herein a new, highly sensitive method for the measurement of H_2_O_2_ and glucose using fluorescence correlation spectroscopy (FCS).

**Methodology/Principal Findings:**

FCS has the advantage of allowing us to determine the number of fluorescent molecules. FCS measures the fluctuations in fluorescence intensity caused by fluorescent probe movement in a small light cavity with a defined volume generated by confocal illumination. We thus developed a highly sensitive determination system of H_2_O_2_ by FCS, where horseradish peroxidase (HRP) catalyzes the formation of a covalent bond between fluorescent molecules and proteins in the presence of H_2_O_2_. Our developed system gave a linear calibration curve for H_2_O_2_ in the range of 28 to 300 nM with the detection limit of 8 nM. In addition, by coupling with glucose oxidase (GOD)-catalyzed reaction, the method allows to measure glucose in the range of 80 nM to 1.5 µM with detection limit of 24 nM. The method was applicable to the assay of glucose in blood plasma. The mean concentration of glucose in normal human blood plasma was determined to be 4.9 mM.

**Conclusions/Significance:**

In comparison with commercial available methods, the detection limit and the minimum value of determination for glucose are at least 2 orders of magnitude more sensitive in our system. Such a highly sensitive method leads the fact that only a very small amount of plasma (20 nL) is needed for the determination of glucose concentration in blood plasma.

## Introduction

A simple and highly sensitive method for the determination of hydrogen peroxide (H_2_O_2_) has broad analytical applications. It is applicable for the measurements of very low levels of H_2_O_2_ in foods, consumer products and environmental waters such as rainwater [1]–[3]. H_2_O_2_ is also present in a variety of biological systems and induces diverse biological effects [4]. For instance, H_2_O_2_ regulates various cellular processes as a signaling molecule whereas its cytotoxic effects are associated with the initiation and progression of many diseases [5]. Because these effects are dependent on the cellular level of H_2_O_2_, the measurement of low concentration of H_2_O_2_ is important for the study on the association of H_2_O_2_ with diseases. In addition, because H_2_O_2_ is generated by an oxidase-mediated reaction, its determination is a basis for the assay of various oxidases and their substrates such as glucose, lactate, glutamate, urate, xanthine, choline, cholesterol and NADPH [1]–[3], [6].

Many methods are now available for the assay of H_2_O_2_ in biological samples. They are spectrophotometry, fluorometry, chemiluminescence and electrochemistry [1]–[3]. Among these methods, horseradish peroxidase (HRP)-catalyzed color and fluorescence reactions have been widely used for the assay of H_2_O_2_ because of their simplicity and high selectivity [1], [2], [7]–[9]. Due to its low background fluorescence, HRP-catalyzed oxidation of 10-acetyl-3,7-dihidroxyphenoxazine (Amplex™ Red) with H_2_O_2_, which forms a highly fluorescent resorfin (λex = 563 nm; λem = 587 nm), has been extensively utilized for the assay of low concentration of H_2_O_2_ in biological samples. However, its detection limit for H_2_O_2_ is 50 nM [1], [10].

Recently, fluorescence correlation spectroscopy (FCS) has been applied to characterize and determine fluorescent components in aqueous solution at nanomolar concentrations [11], [12]. FCS measures the fluctuation in fluorescence intensity caused by diffusion of fluorescent components through a small light cavity with a confocal detection volume (0.25 fL), and analysis of the fluorescence fluctuation offers information on mobility and concentration of fluorescent components in sample solution. When a fluorescent probe of low-molecular weight binds to protein in sample solution, slow-diffusing component (protein labeled with fluorescent probes) increases with the decrease in fast-diffusing component (fluorescent probe), which affects the fluorescence autocorrelation curve in FCS. The fluorescence autocorrelation curve is obtained after the fluctuations are recorded as a function of time and statistically analyzed by autocorrelation analysis. The average residence time (τ) and the absolute numbers of slow- and fast-diffusing components in the small volume can be deduced by the fluorescence autocorrelation function calculated from the fluorescence autocorrelation curve.

**Figure 1:**
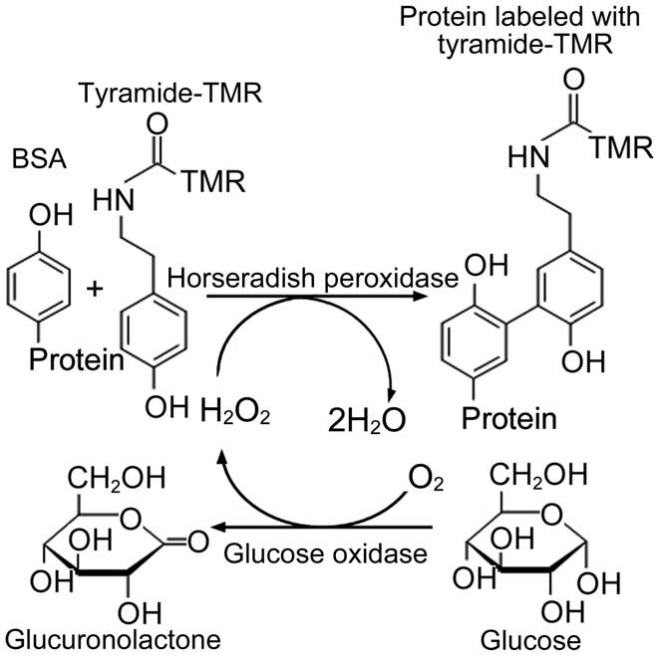
Scheme of the enzymatic reaction. This scheme shows binding between tyramide labeled with tetramethyl rhodamine (TMR) and proteins by peroxidase and H_2_O_2_. Because the H_2_O_2_ concentrations correspond to those of the proteins labeled with tyramide-TMR, we can determine the concentrations of H_2_O_2_ by FCS. Further, because H_2_O_2_ is produced by the reaction between glucose and glucose oxidase, we can determine the concentrations of H_2_O_2_ and thus deduce the concentrations of glucose.

In order to develop a highly sensitive method for the assay of H_2_O_2_ by FCS, we used tyramide labeled with tetramethyl rhodamine (tyramide-TMR) as a fluorescent probe and bovine serum albumin (BSA) as a protein. Tyramide labeled with fluorescent probes have been utilized as reporter fluorescent substrate for HRP-catalyzed deposition (CARD) that is signal amplification technique in immunoassay and in situ hybridization of nucleic acids [13], [14]. Similarly to tyramine and tyrosine [9], [15], [16], the tyramide gives 2,2′-dihydroxydiphenyl derivatives *via* tyramide radical, in the HRP-catalyzed oxidation with H_2_O_2_ when it is present at high concentrations. However, when applied at lower concentrations in the presence of BSA, tyramide radical binds to an electron-rich moieties of BSA such as a tyrosine residue, to give TMR-labeled BSA [14], as shown in Figure 1. Under the optimized conditions, TMR-labeled BSA (fraction of slow-diffusing component) measured by FCS was found to be proportional to H_2_O_2_ concentration of sample solution from 28 nM to 300 nM with a detection limit of 8 nM (S/N = 3). By coupling with glucose oxidase (GOD)-mediated reaction, the present method was applicable to selective assay of glucose (Figure 1) with a detection limit of 24 nM, which enabled to assay glucose in human plasma with only a very small amount of plasma (20 nL).

## Methods

### Reaction solution for H_2_O_2_ determination

The reaction solution consisted of 0.001% P-10, 10 nM tyramide-TMR (Perkin Elmer, Waltham, MA, USA), 2 mg/mL BSA, and 3 U/mL HRP (Roche, Basel, Switzerland) in 100 mM Tris buffer (pH 7.5). Here, P-10 is a detergent for the suppression of binding between tyramide-TMR and reaction tubes. The P-10 was kindly provided by Tauns, Numazu, Japan. To the reaction solution of 480 µL, 20 µL of various concentrations of H_2_O_2_ solution (0, 0.2, 0.25, 0.5, 2.0, 2.5, 5.0, 7.5, and 12.5 µM) was added and incubated in a chambered coverslip (Lab-Tek 155411; Nalge Nunc International, Roskilde, Denmark) of FCS at room temperature for 20 min. For each concentration of the H_2_O_2_ solution, the measurements by FCS were performed 3 times.

### Reaction solution for glucose determination

The reaction solution consisted of 0.001% P-10, 10 nM tyramide-TMR, 2 mg/mL BSA, 5 U/mL HRP, and 5 U/mL GOD (Roche) in 100 mM Tris buffer (pH 7.5). To the reaction solution (480 µL), 20 µL of various concentrations of glucose solution (0, 0.50, 0.75, 1.25, 2.50, 10.0, 20.0, 25.0, 30.0, 37.5 and 50.0 µM) was added and incubated in a chambered coverslip of FCS at room temperature for 30 min. For each concentration of the glucose solution, the measurements by FCS were performed 3 times.

### Determination of glucose in human blood plasma

The reaction solution was the same as that for glucose as described above. Human blood plasma was purchased from George King Bio-Medical (Cat No. 0020; Overland Park, KS, USA). The plasma solutions were prepared by dilution with distilled water by a factor of 200, 400, 500, 1000, 1600 and 2000. The reaction solution (480 µL) and the diluted plasma solution (20 µL) were mixed and incubated in a chambered coverslip of FCS at room temperature for 30 min. For each concentration of the plasma solution, the measurements by FCS were performed 3 times.

### FCS measurement

The FCS used here was NovousGene Compact FCS (NG1532, Hamamatsu Photonics, Hamamatsu, Japan), which consisted of an LD-excitation solid-state laser (maximum power = 1 mW) and a water immersion objective (40×, NA = 1.15; Olympus, Tokyo, Japan). The excitation wavelength was 532 nm, and emissions were detected at 565 nm. The confocal pinhole diameter was adjusted to 25 µm. The sample volume was 500 µL. The diffusion time (τ_free_) of tyramide-TMR was obtained from the experiments with 0 µM H_2_O_2_ solution or 0 µM glucose solution, and this diffusion time was fixed throughout the FCS measurements. The diffusion time (τ_bound_) of tyramide-TMR-BSA was obtained from the experiments with 12.5 µM H_2_O_2_ solution or 50.0 µM glucose solution, and this time was also fixed throughout the FCS measurements. For experiments with human plasma, both the diffusion time (τ_free_) of tyramide-TMR and the diffusion time (τ_bound_) of tyramide-TMR-BSA were set to be the same as the values for glucose solution. The FCS data were analyzed by software equipped with the FCS (Hamamatsu Photonics).

### Colorimetric determination of H_2_O_2_ and glucose with Amplex™ Red

As a commercial available method for comparison with the present method, we used the Amplex™ Red glucose/glucose oxidase assay kit (A22189; Molecular Probes, Eugene, OR, USA) for measurements of H_2_O_2_ and glucose. The measurements were performed according to the manufacturer's manual. We measured the absorbance of 571 nm using a multimode microplate reader (SpectraMax M5TB, Molecular Devices, Sunnyvale, CA, USA). For each concentration of H_2_O_2_ solution or glucose solution, the measurements with Amplex™ Red were performed 3 times.

### Detection limit and minimum value of determination

All the experimental data were obtained by subtracting the mean value of blank signals from each of the corresponding measured data. The limit of detection was obtained by 3×(standard deviation of blank signals)/(slope of a regression line for experimental data), and the minimum value of determination was obtained by 10×(standard deviation of blank signals)/(slope of a regression line for experimental data).

## Results

### Determination of H_2_O_2_ concentrations

**Figure 2:**
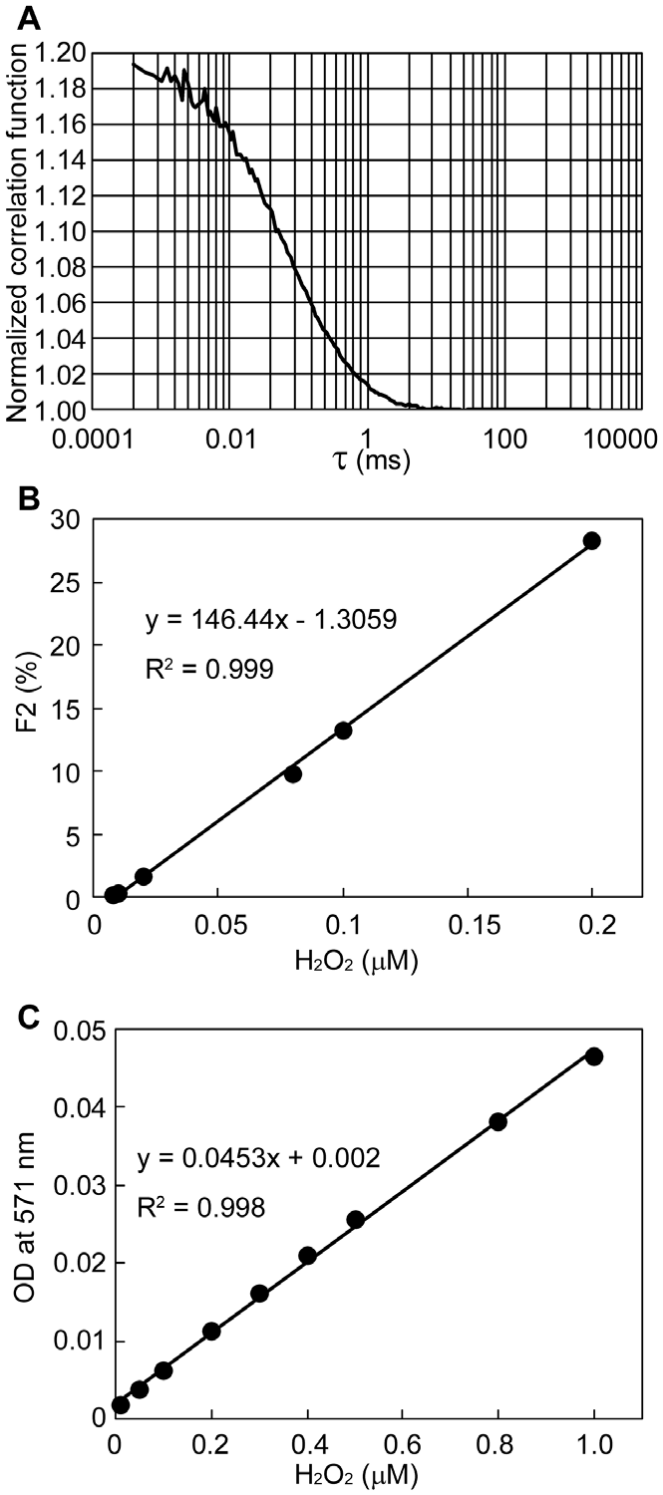
Determination of H_2_O_2_ concentration. A. FCS curve proves that our FCS measurements are successful for the detection of 0.2 µM H_2_O_2_. B. Calibration curve by an FCS method. F2 indicates the existence ratio for tyramide-TMR-BSA. The SD values are too small to set the error bars in this figure. For example, when the H_2_O_2_ concentration was 0.008 µM, the average value of F2 was 1.2% and the SD value was 1.0%. When the H_2_O_2_ concentration was 0.2 µM, the average value of F2 was 29.1% and the SD value was 2.8%. C. Calibration curve by the Amplex™ Red method. The measurements by FCS and Amplex™ Red were performed 3 times each.

In the FCS experiments, when the H_2_O_2_ concentrations were increased, the existence ratio for tyramide-TMR decreased while that for tyramide-TMR-BSA increased. The former existence ratio was referred to as F1, and the latter was F2. The FCS curve proved that our FCS measurements were successful (Figure 2A). As shown in Figure 2B, a linear relation between H_2_O_2_ concentration and F2 was obtained in the range of 28–300 nM H_2_O_2_ with the detection limit of 8 nM. In a commercial available method using Amplex™ Red, we found that the detection limit was 19 nM and that the range of determination was 65 nM–1 µM (Figure 2C). That is, the detection limit and the minimum value of determination for H_2_O_2_ in our system were slightly more sensitive than by the commercial available method.

### Determination of glucose concentrations

**Figure 3:**
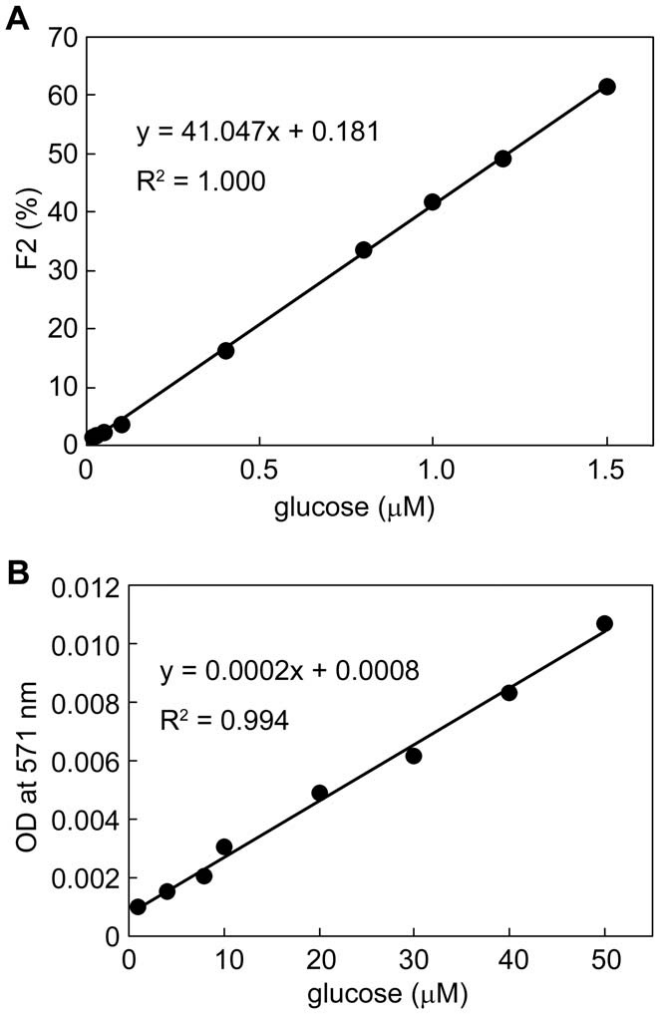
Determination of glucose concentration. A. Calibration curve by an FCS method. F2 indicates the existence ratio for tyramide-TMR-BSA. The SD values are too small to set the error bars in this figure. For example, when the glucose concentration was 1.0 µM, the average value of F2 was 41.8% and the SD value was 1.3%. When the glucose concentration was 1.5 µM, the average value of F2 was 61.4% and the SD value was 2.4%. B. Calibration curve by the Amplex™ Red method. The measurements by FCS and Amplex™ Red were performed 3 times each.

The determination of β-D-glucose is important in the area of industrial quality control and processing applications as well as in clinical diagnosis and treatment of diabetes [17]–[19]. Although various methods for the determination of glucose have been reported, enzymatic ones using GOD have been widely used due to their simplicity and selectivity. Therefore, we attempted to develop a highly sensitive method for the determination of glucose by coupling our FCS method with GOD-catalyzed oxidation of glucose (Figure 1). Under the optimized conditions, a linear relation between glucose concentrations and F2 (i.e. existence ratio for tyramide-TMR-BSA) was obtained in the range of 80 nM–1.5 µM glucose with the detection limit of 24 nM as shown in Figure 3A. In contrast, a commercial available method using Amplex™ Red showed a limit of detection of 2 µM and a range of determination of 6 µM–50 µM (Figure 3B). That is, the detection limit and the minimum value of determination for glucose in our system were at least 2 orders of magnitude more sensitive than in the commercial available method.

### Determination of glucose concentrations in human blood plasma

**Figure 4:**
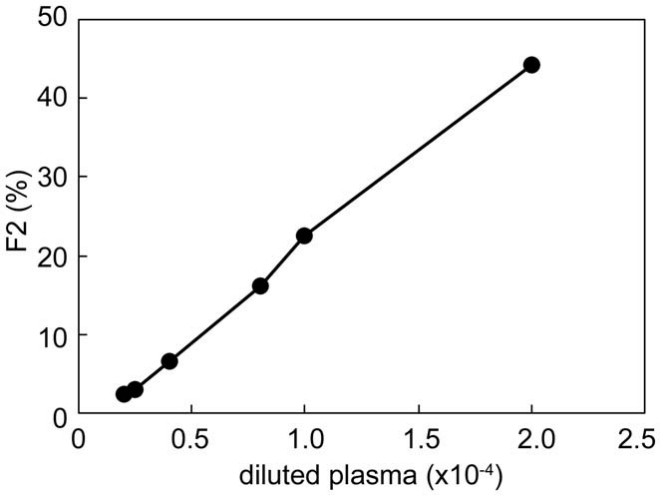
Determination of glucose concentration in human blood plasma. The measurements by FCS were performed 3 times. The F2 values obtained here were calibrated by Figure 3A, and then the glucose concentrations were calculated by taking account of dilution.

The relation between dilution factor of plasma and F2 is shown in Figure 4. The limit of detection was obtained by use of the diluted plasma of 0.2×10^−4^. The range of determination was between the diluted plasma of 0.4×10^−4^ and that of 2.0×10^−4^. We thus applied the F2 values obtained from the diluted plasma of 0.4×10^−4^, 1.0×10^−4^ and 2.0×10^−4^ to the calibration curve of Figure 3A, and calculated the glucose concentrations by taking account of dilution. As a result, the glucose concentrations in human blood plasma were estimated to be 4.0 mM, 5.4 mM and 5.4 mM for the diluted plasma of 0.4×10^−4^, 1.0×10^−4^ and 2.0×10^−4^, respectively. The average value of 4.9 mM is in good agreement with the data (3.9–6.1 mM) that are presented in a textbook of physiology [20]. Further, we asked a clinical laboratory examination company (Shikoku Chuken, Ayagawa, Kagawa, Japan) to measure the glucose concentration of our sample and obtained 4.6 mM, supporting that our data were reasonable. However, it is noteworthy that our method needs only 20 nL plasma for this determination. That is, only about 40 nL is needed as a blood sample. In contrast, when we used the Amplex™ Red method, the glucose concentrations were obtained to be 67 mM and 158 mM for the diluted plasma of 0.5×10^−4^ and that of 5.0×10^−4^, respectively. The values seem incorrect (see Discussion).

## Discussion

A current priority is the development of simple and rapid methods that facilitate reproducible determination of various species at low concentrations [21]. In the present study, we applied FCS to detect and determine H_2_O_2_ and glucose at low concentrations. Our developed system using FCS is versatile because of its use of a commercially available fluorescent probe, but not radio isotopes or other special chemicals. We thus succeeded in determining glucose concentrations by the detection of H_2_O_2_ generated from the GOD-catalyzed oxidation of glucose. The detection limit and the minimum value of determination for glucose were found to be at least 2 orders of magnitude more sensitive than with the commercial available method. We should note that not only BSA (2 mg/mL = 3.0×10^−8^ mol/mL) but also HRP (5 U/mL = 3.6×10^−11^ mol/mL) and GOD (5 U/mL = 1.6×10^−10^ mol/mL) were included in the reaction solution with tyramide-TMR for the FCS measurements. However, the reaction involved in both HRP and GOD can be neglected because the molar ratio of (HRP and GOD)/BSA was <0.0065. That is, the fluorescence signals detected by FCS were thought to mainly originate from free tyramide-TMR and tyramide-TMR-labeled BSA.

At present, Amplex™ Red is widely used for the detection of H_2_O_2_ [10]. However, the use of Amplex™ Red offered us the extraordinary data of glucose concentration in human blood plasma (e.g. >tens of mM). This fact might be caused due to the reaction between Amplex™ Red and some molecules in blood plasma. For example, there seem to be some inhibition in the HRP reaction by reducing agents like ascorbate and cysteine in human blood. We do not know the exact reason but this inhibition is well canceled in our FCS method. Therefore, our developed method is completely superior to the measurement by use of Amplex™ Red. Moreover, our method is thought to be useful to detect the concentration of other molecules in organisms if the corresponding oxidase is available.

We should note that we need only a small amount of blood (i.e. tens of nano litter) to determine the glucose concentration in blood. This fact gives two advantages. The first one is that our method will offer the biopsy that reduces a burden to patients. For example, when using alcohol oxidase, we will detect the blood alcohol concentration. The use of ascorbate oxidase will detect the ascorbate concentration in foods; the use of cholesterol oxidase will detect the blood cholesterol concentration for medical checkup; the use of choline oxidase will detect the blood choline concentration for mental condition check; and the use of pyruvate oxidase will detect the blood pyruvate concentration for freshness check of preserved blood [1]–[3], [6]. The second one is that our method will contribute to facilitate medical and biological researches at a single-cell level. Because the contents in cells do not prevent from determination of a target molecule in our FCS method, the second advantage will be realized in the near future.
